# Unbecoming Medical Journalism

**Published:** 1869-07

**Authors:** 


Unbecoming Medical Journalism.—The June number of the *Nashville Journal of Medicine and Surgery*, contains an article of abuse and scandal, by T. S. Bell, M. D., which is too low and vindictive for publication in any paper, unless conducted in the exclusive interests of roughs and blackguards. The immediate provocation was the review of a work written by Dr. Bell, which appeared in the *Richmond and Louisville Medical Journal*, from the pen of the editor, Prof. E. S. Gaillard.

